# Psychometric properties of the Spanish version of the European Organization for Research and Treatment of Cancer Quality of Life Questionnaire (EORTC QLQ-C30)

**DOI:** 10.1007/s11136-021-03068-w

**Published:** 2021-12-20

**Authors:** Caterina Calderon, Pere J. Ferrando, Urbano Lorenzo-Seva, Estrella Ferreira, Eun Mi Lee, Marta Oporto-Alonso, Berta M. Obispo-Portero, Luka Mihic-Góngora, Adan Rodríguez-González, Paula Jiménez-Fonseca

**Affiliations:** 1 Department of Clinical Psychology and Psychobiology, Faculty of Psychology, University of Barcelona, Passeig de la Vall d’Hebron, 171, 08035 Barcelona, Spain; 2 Department of Psychology, Faculty of Psychology, Rovira and Virgili University, Tarragona, Spain; 3 Department of Psychology, University Abat Oliba, Barcelona, Spain; 4 Department of Medical Oncology, Hospital Universitario Infanta Leonor, Madrid, Spain; 5 Department of Medical Oncology, Hospital Universitario Central of Asturias, Oviedo, Spain

**Keywords:** Quality of life, Psychological distress, Oncology, Psychometric, Invariance

## Abstract

**Purpose:**

The aim of this study was to analyze the internal structure of the EORTC QLQ-C30, to examine the validity and normative data for cancer patients.

**Method:**

Exploratory and Confirmatory factor analyses were conducted to explore the scale’s dimensionality and test for strong measurement invariance across sex and tumor site. All the analyses were based on a multicenter cohort of 931 patients who completed the Brief Symptom Inventory (BSI-18) and the EORTC QLQ-C30.

**Results:**

Our findings indicate that the EORTC QLQ-C30 has acceptable psychometric properties and an internal structure that is well accounted for a bifactor model: a general factor that evaluates quality of life and a group factor that would analyze physical health that would be defined by physical function, role function, and fatigue. The result of the multi-group CFA revealed a strong invariance according to sex, tumor, and over time. Reliability of the EORTC exceeding 0.86 and the simple sum of the items of the scale was a good indicator of oncology patients’ quality of life. Both factors correlate closely with depression, anxiety, and psychological distress and are sensitive to change, especially the quality of life, with a significant decrease in the post-test.

**Conclusion:**

The Spanish version of the EORTC QLQ-C30 proved to be a valid, reliable instrument to appraise quality of life in cancer patients. The normative data collected from this study may be useful for the early detection of initial symptoms of deterioration of quality of life in oncology patients.

## Plain English summary

The aim of this study was to analyze the internal structure of the EORTC QLQ-C30, to examine the validity and normative data for cancer patients. Exploratory and Confirmatory factor analyses were conducted to explore the scale’s dimensionality and test for strong measurement invariance across sex, and tumor site. All the analyses were based on a multicenter cohort of 931 patients who completed the Brief Symptom Inventory (BSI-18) and the EORTC QLQ-C30. Our findings indicate that the EORTC QLQ-C30 has acceptable psychometric properties and an internal structure that is well accounted for a bifactor model: a general factor that evaluates quality of life and a group factor that would analyze physical health and that would be defined by physical function, role function and fatigue. The result of the multi-group CFA revealed a strong invariance according to sex, tumor and over time. Reliability of the EORTC exceeding 0.86, and the simple sum of the items of the scale was a good indicator of oncology patients’ quality of life. Both factors correlate closely with depression, anxiety, and psychological distress and are sensitive to change, especially the quality of life, with a significant decrease in the post-test. The Spanish version of the EORTC QLQ-C30 proved to be a valid, reliable instrument to appraise quality of life in cancer patients. The normative data collected from this study may be useful for the early detection of initial symptoms of deterioration of quality of life in oncology patients.

## Background

The EORTC QLQ C30 questionnaire was developed by the European Organization for Research and Treatment of Cancer (EORTC) more than 25 years ago as a system by means of which to measure quality of life in patients who participated in international clinical trials [1]. Its translation into more than 82 languages (https://qol.eortc.org) has made it one of the most widely used instruments to assess health-related quality of life [2–5]. Cancer and its treatment side effects are associated with diminished quality of life for the patient [1]. Quality of life evaluations provide valuable information for the oncologist when choosing anti-tumor therapy and the support measures the patient will require [6] and is one of the aspects most impacted by the tumor and treatment side effects [1].

The EORTC QLQ-C30, version 3, comprises 30 items scored using a Likert-type format; all the items have four levels, except for the last two that have seven levels. The scale relies on a 1-week timeframe for appraisal. The 30 items are distributed into five functional, three symptom subscales, six additional individual items, and a two-item overall subscale. All told, it measures 15 quality-of-life domains [1]. The score is linearly transformed on a scale from 0 to 100 [1].

The EORTC-QLQ-C30 has proven to be reliable and valid in a range of patient populations and in a variety of treatment settings [2, 5, 7]. Internal consistency estimates (Cronbach α coefficient) for the scale scores exceed 0.70 [2, 4]. Test–retest reliability coefficients range between 0.80 and 0.90 for most of the scales with several elements and for the single-item scales [2, 4]. Validity testing have evidenced that the scale provides significant differences between patients with localized cancer vs those with metastatic disease, patients in active treatment vs follow up, and is sensitive to clinical status over time [7–9].

To date, there have been a limited number of studies that have examined the dimensionality and structure of the EORTC QLQ C30, and most have used relatively small samples. Some of these studies have relied on the classical test theory-based, purely exploratory techniques intended for use at scale level [10–12] and can only provide preliminary information. Other studies [10–13] have applied multi-trait scaling analyses, which yield useful external validity evidence (prediction or relevant external variables or known-group outcomes), but no information regarding the internal properties of the measure. Finally, studies based on exploratory factor analysis (EFA) at the item level have achieved heterogeneous results. The original model is designed to generate 15 dimensions and three general indices: functioning scale, symptom scale, and the global health scale [14, 15]. Others have replicated the standard model and obtained an empirical solution with 5 domains [16]. Some authors have devised a two-bidimensional model of *physical health* and *mental health* [17]. Still other authors assess the scale as a single dimension, assuming that all first order latent variables (except QL) would load onto a single underlying dimension [18].

The initial data regarding the properties of the Spanish version of the EORTC QLQ C30 in a sample of 137 patients with prostate cancer have been analyzed by multi-trait scaling [19]. Consequently, they have gleaned useful external validity evidence, but no internal evidence regarding the dimensionality, structure, and accuracy of the derived scores in our context, leaving these properties inadequately examined. The aim of this study is therefore to: (1) analyze the psychometric properties of the EORTC QLQ-C30 scale, notably, dimensionality, structure, and score accuracy; (2) explore the invariance of the measure and differences in level in groups defined by sex and tumor site; (3) provide evidence of score validity; 4) gauge sensitivity to the change brought about by treatment, and (5) generate updated normative data of the scale in a sample that is representative of the adult Spanish population with cancer*.*

## Method

### Instruments

The EORTC QLQ-C30 version 3.0 [1] includes 30 items comprising five multi-item functional scales: physical (PF), role (RF), cognitive (CF), emotional (EF), and social (SF); 3 multi-item symptom scales: fatigue (FA), nausea and vomiting (NV), and pain (PA); 6, single-item additional symptoms commonly reported by cancer patients: dyspnea (DY), insomnia (SL), appetite loss (AP), constipation (CO), and diarrhea (DI), as well as the perceived financial impact (FI) of the disease and treatment, and a two-item global health status/QoL scale. The questionnaire uses a 1-week time frame and 4-point Likert-type response scales (“not at all,” “a little,” “quite a bit,” and “very much”), with the exception of the two items of the overall QL scale, which are rated 1–7. The Spanish version of EORTC QLQ-C30 presented by the EORTC was used for data collection. A sample can be downloaded at the following URL: http://groups.eortc.be/quol/erotc-qlq-c30. Following standard European Organization for Research on the Treatment of Cancer procedures, all scores were linearly converted into a 0 to 100 scale, with higher scores indicating a higher level of functioning, better HRQOL for global health status, and more severe symptoms [1]. It was administered to the patients prior to initiating chemotherapy and, again, 6 months later.

The *Brief symptom Inventory* (BSI-18) consists of 18 items categorized into three dimensions (depression, anxiety, and psychological distress) and rated on a five-point scale [20] and has been adapted to Spanish cancer patients [21]. Raw scores are converted to *T*-scores based on sex-specific normative data. Alpha coefficients were between 0.75 and 0.88 for the Spanish version among cancer patients [21].

Patient and tumor characteristics were obtained from the interview and clinical history. The following variables were compiled: sex, age, marital status, education level, employment status, tumor site, stage, and treatment.

### Procedure

**Fig. 1:**
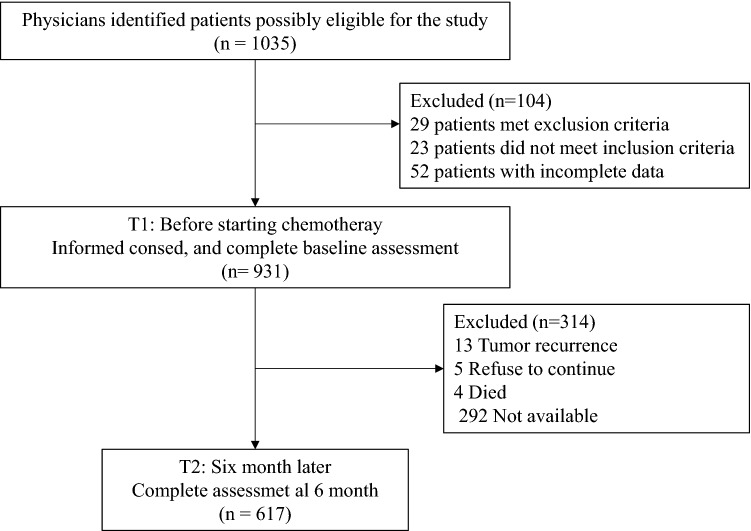
Participant flow chart

The data are derived from a prospective cohort of patients with non-metastatic colon cancer from the multicohort NEOCOPING study promoted by the Continuous Care Group of the Spanish Society of Medical Oncology (SEOM) and conducted in 17 Spanish medical oncology departments. Individuals > 18 years of age with resected, non-metastatic colon cancer and eligible for adjuvant chemotherapy were consecutively enrolled. Those who had received preoperative chemotherapy or radiotherapy, and those with any condition that impeded comprehension of or participation in the study were excluded. Patients completed the questionnaires at home and returned them at the following appointment for adjuvant cancer treatment to be initiated. Subjects were followed until adjuvant treatment was ended and questionnaires were filled out again after approximately 6 moths. The flowchart comprising inclusion and exclusion criteria of participants is given in Fig. 1.

Ethical approval was obtained by the Institutional Research Ethics Committee of each hospital and the Spanish Agency for Medicines and Health Products (AEMPS) (number L34LM-MM2GH-Y925U-RJDHQ); informed consent for voluntary participation was obtained in writing from all subjects prior to performing any study procedure. STROBE guidelines were used to ensure the reporting of this study [22].

### Data analysis

A five-stage approach was used in the analyses. First, the dimensionality and structure of the EORTC were assessed in the entire sample through exploratory factor analysis. To evaluate dimensionality, we computed parallel analysis (PA); [23] and essential unidimensional indices [24]: unidimensional congruence (Unico), explained common variance (ECV), and mean of item residual absolute loadings (MIREAL). PA compares the dimensions obtained in the sample dataset to the ones obtained in random datasets in which the null model holds: the number of dimensions in the sample dataset that account for more common variance than the ones based on random datasets is the number of factor advised to be extracted in the sample dataset. The indices to assess essential unidimensionality help to decide whether a strong, dominant factor exists: when the value of the three indices computed crosses their particular threshold, the unidimensional factor solution is advised. The threshold values that determine that the factor solution is essentially unidimensional were: UniCo > 0.95, ECV > 0.85, and MIREAL < 0.30. As the outcome of these indices were inconclusive, we computed an exploratory bifactor model to decide between a single-factor model and a two-factor model [25]. The bifactor model allows the hypothesis of a general factor, while addition common variance is modeled using group factors. When a formal hypothesis does not exist for the overall bifactor model (i.e., the general factor and a particular number of group factors), Pure Exploratory Bifactor models (PEBI) can be inspected [25]. Second, inasmuch as a clear, cross-validation-resistant, factorial structure was finally identified in the first stage, multiple-group confirmatory factor analyses (CFAs) were performed to gauge measurement invariance in groups defined by gender and tumor localization. Third, the reliability and appropriateness for individual assessment of the scores derived from the chosen FA solution were examined using a multi-faceted approach [26]. Fourth, the sensitivity of treatment-induced change was assessed by fitting two-wave (pretest, posttest) structural equation modeling. Finally, convergent validity was appraised by means of a full structural model in which the CFA was extended to include external variables.

## Results

### Participants

The total sample of this study comprises 931 cancer patients (569 women; 61%). Participants ranged from 24 to 85 years of age (*M* = 58.9 years, SD = 12.2). Most were married or partnered (75.9%) and had a primary level of education (53.9%); 58.6% were employed. The most prevalent cancer types were colon (42.2%) and breast (34.4%), stage I-II (56.4%), and scheduled for treatment with adjuvant chemotherapy and radiotherapy (66.5%). A total of 617 patients (66.3%) participated in a second testing session (261 women; 42.3%) 6 months after the first one. No significant differences were detected regarding EORTC scale scores between the group that participated in the retest and those that did not.

### Descriptive statistics for EORTC items

Table 1 shows the means and standard deviations of the Spanish version of QLQ-C30 (version 3.0) administered pre-chemotherapy and 6 months later. Mean item scores ranged from 77.1 (social) to 86.0 (physical) on the functional scales. The functional scale score distribution displayed negative asymmetry on all the scales: scores often exceeded 70.0 (that is, many patients exhibited acceptable functioning overall). The symptom scales and the single-item measures reveals a positive asymmetrical distribution, with scores close to 0 (no symptoms), except for insomnia and fatigue with mean scores of 30.1 and 20.0, respectively.

**Table 1:** Descriptive statistics and scale reliability of the EORTC QLQ-C30

	Items^a^	Pre-chemotherapy		6 months later	
		Mean score	SD	Mean score	SD
Functional scales^b^		79.4	16.6	78.7	19.4
Physical	1–5	86.0	15.1	83.4	18.0
Role	6, 7	76.4	26.1	75.5	27.5
Emotional	21–24	72.1	24.7	75.7	25.2
Cognitive	20, 25	85.6	20.3	82.5	23.1
Social	26, 27	77.1	25.3	76.2	27.0
Symptom scales^c^		18.1	14.5	20.0	17.2
Fatigue	10, 12, 18	29.4	24.3	36.0	27.6
Nausea and vomiting	14, 15	9.1	18.3	11.2	20.2
Pain	9, 19	17.9	23.3	20.3	26.3
Dyspnea	8	5.0	15.9	6.8	19.1
Sleep disturbance	11	31.3	32.3	32.0	33.3
Appetite loss	13	20.2	29.5	20.7	30.3
Constipation	16	21.0	30.1	23.3	31.3
Diarrhea	17	13.8	24.4	16.0	26.5
Financial impact	28	15.4	28.1	14.0	27.6
Health status	29, 30	69.8	20.3	67.2	24.1

^a^Numbers correspond to the item numbers in the questionnaire

^b^Scores range from 0 to100; higher scores represent a higher level of functioning or global health status

^c^Scores range from 0 to 100 with a higher score representing a greater symptom severity

### EORTC dimensionality assessment

The EORTC items are ordered-categorical with only four response points and, in this study, were administered to a large sample. Furthermore, the results of the previous section revealed that the distribution of some items is greatly skewed in both directions. In this scenario, the non-linear item factor analysis model, based on an underlying variables approach, (see e.g., [27]) was deemed the most suitable to fit the EORTC data. To fit this model, we followed the procedure described in [28]: the inter-item correlation to be analyzed was the polychoric correlation matrix, and was fitted using the robust unweighted least squares method. This general setting was applied in all structural analyses conducted in this study (EFAs, CFAs, two-wave, and extended validity model) and proved feasible in all cases. EFAs were performed using the FACTOR^1^ software [29]. The remaining analyses were conducted using Mplus version 5.1 [30].

The data were found to be appropriate to fit the EFAs (and subsequent analyses). The KMO index (0.810) and Barlett’s test (*χ*2 = 10.582.3, df = 435, *p* < 0.001) suggested that there was enough inter-item consistency to fit the FA model.

PA results suggested a two-factor solution as the most suitable for the sample data. However, when a single factor was extracted, goodness-of-fit indices indicated that the model was almost marginally acceptable (RMSEA = 0.072; CFI = 0.970; GFI = 0.956). Furthermore, the values of indices assessing essential unidimensionality (Unico = 0.956; ECV = 0.840, and MINREAL = 0.217) suggested that the single-factor solution is close to essential unidimensionally, but not perfectly, and that fitting more than one dimension is advisable. The situation in which a set of items is essentially unidimensional, but in which a (generally small) subgroup of items share specific variance, is relatively common in practice. This alternative model can be examined using an exploratory bifactor model (PEBI). The outcome of PEBI in our sample data is presented in Table 2.

**Table 2:** Bifactor loading matrix of the EORTC QLQ-C30

Items		Quality of life	Physical health
1	Physical function	**0.468**	**0.508**
2	Physical function	**0.479**	**0.623**
3	Physical function	**0.480**	**0.525**
4	Physical function	**0.506**	**0.423**
5	Physical function	**0.374**	**0.408**
6	Role function	**0.597**	**0.520**
7	Role function	**0.565**	**0.524**
8	Dyspnea	** − 0.569**	0.150
9	Pain	** − 0.593**	0.265
10	Fatigue	** − 0.627**	** − 0.429**
11	Insomnia	** − 0.566**	0.012
12	Fatigue	** − 0.718**	0.348
13	Appetite loss	** − 0.583**	0.242
14	Nausea and vomiting	** − 0.485**	0.341
15	Nausea and vomiting	** − 0.373**	0.302
16	Constipation	** − 0.398**	0.090
17	Diarrhea	** − 0.356**	0.245
18	Fatigue	** − 0.688**	** − 0.415**
19	Pain	** − 0.679**	0.321
20	Cognitive functioning	**0.669**	0.083
21	Emotional functioning	**0.773**	0.369
22	Emotional functioning	**0.771**	0.327
23	Emotional functioning	**0.745**	0.231
24	Emotional functioning	**0.831**	0.205
25	Cognitive functioning	**0.505**	0.111
26	Social functioning	**0.695**	0.185
27	Social functioning	**0.709**	0.319
28	Financial difficulties	**0.440**	0.072
29	Global health status	**0.476**	0.354
30	Global health status	**0.535**	0.317

Loading on the general factor and in the group factor (if > 0.40) are printed in bold

As depicted in the table, all the items in the scale have a substantial loading value in the general factor (Quality of Life). Moreover, the 5 items related to physical function, the 2 items concerning role function and 2 items addressing symptoms of fatigue define a group factor (Physical Health, loading values > 0.40). This factor combines aspects connected to physical status and fatigue-related symptoms, and could be sensitive to treatment toxicity. As for model data fit, goodness-of-fit indices suggested that the model could now be deemed acceptable (RMSEA = 0.053; CFI = 0.985; GFI = 0.977).

The stability and replicability of the proposed solution, as well as its appropriateness for a confirmatory solution, were examined using a double cross-validation schema. Once the sample had been randomly split into two halves, the factorial congruences between the obtained solution in each half sample were always above 0.98 for both the general and group factors. Furthermore, when a bifactor CFA solution was specified in the second half sample, based on the exploratory bifactor solution obtained in the first half, the specified CFA solution was always the same and fitted well. Given these results, a confirmatory bifactor solution in which the general factor was defined by all the EORTC items and the group factor (physical health) was defined by the 9 items described above was considered the most appropriate for the entire group, and served as the basis for all the subsequent CFA analyses.

### Multiple-group confirmatory factor analyses and measurement accuracy

Based on the common CFA solution described above, strong invariance multiple-group solutions were fitted using sex and tumor site as grouping variables. As discussed in detail elsewhere [21], if strong measurement invariance is achieved, it can be assumed that the same two factors are measured in the different groups and that the EORTC items function in the same way in these groups. Thus, differences in mean group scores can be validly interpreted as reflecting ‘true’ group differences in the general and group dimensions being measured. The strong invariance solution was identified by using the delta parameterization for the residuals (see [30]) and fixing the factor means and variances to zero and 1, respectively, in the first group. Preliminary, weaker-invariance solutions (non-invariant loadings, thresholds, or both) were tried and we found that the strong solution fitted better in relative terms (as indicated by the RMSEA) and also according to the parsimony indices (the Bayesian information criterion). Because the strong solution also fitted acceptably well in overall terms (see Table 3), we only report the group estimates derived from it, which are displayed in Table 3.

**Table 3:** Results of the strong invariance model for sex and tumor site

Groups	Means		CFI	RMSEA	(90% CI)
	QoL	Physical			
Sex			.95	.060	(.058; .063)
Men (fixed)	.00	.00			
Women	.05	− .06			
Tumor			.94	.061	(.058; .064)
Colon (fixed)	.00	.00			
Breast	− .35*	.19*			
Others	− .40*	− .25*			

*CFI* Comparative Fit Index; *RMSEA* root mean square error of approximation

*Significantly different from zero at the .05 level (two-tailed)

So as to interpret the mean differences in the table and for identification purposes, the means are always fixed to zero in the first group and are freely estimated in the remaining. The results can be summarized as follows. First, no mean differences in any of the factors (Quality of Life and Physical Health) were detected by sex. Second, mean differences in both factors were found for tumor location. This might be due to the fact that there are patients with chemotherapy regimens that entail more side effects than other and with the possible effect of surgery prior to treatment, given that the sample consisted of individuals with different types of tumors.

As the results uphold the assumption that the EORTC items function in the same way in both groups, accuracy and appropriateness measures of the EORTC scores can be obtained for the whole group. First, marginal reliability measures were attained for two types of scores: (a) factor score estimates obtained by using all the available information in the CFA solution and (b) the simplest raw scores achieved by adding all the EORTC items (Quality of Life scale) and the salient items in the group factor (Physical Health scale). Reliability estimates for the factor scores were 0.96 (QoL) and 0.88 (Physical Health). The corresponding reliability estimates (omega coefficients) for the raw scores were 0.94 and 0.86. Hence, for both factors, all the scoring schemas are highly reliable, particularly for the scores intended to quantify the general factor. As expected, the most informative factor score estimates are more reliable than raw scores, albeit the latter can already be considered reliable enough to be used in clinical assessment. To assess this issue further, the ordinal coefficients of fidelity were also computed for the raw scores. These coefficients estimate the correlation between each set of raw scores and the factor they intend to measure [31]. Fidelity estimates were 0.97 (QoL) and 0.91 (Physical Health). To sum up, QoL and Physical Health scale scores can be obtained for the EORTC items and these scores are both accurate and representative of the factors they seek to measure.

### Two-wave CFA model to gauge treatment-induced change

The bifactor CFA solution used in the previous section was extended to a two-wave panel model (Pretest, Posttest) using similar constraints as those in the previous multi-group analyses. More specifically, strong invariance was imposed here but, instead of across groups, it was over time. If this restriction is found to be acceptable, inasmuch as the items function with the same properties at both time points, mean group changes in both factors can be validly and univocally assessed. Thus, strong invariance over time was assumed and the mean of each dimension at Time 1 was set to zero. Results are illustrated in Table 4 and can be summarized as follows. First, the models exhibited an acceptable fit. Second, significant pretest–posttest changes were observed for both factors, especially the QoL factor, that consisted of decreased group means on posttest measures. While these changes are highly significant (given the high power of the test), they would be qualified as small in terms of effect sizes (Cohen’s d).

**Table 4:** Results of the two-wave model to assess change

	Means		CFI	RMSEA	(90% CI)
Dimension	Time 1	Time 2	.950	.042	(.040; .044)
Quality of life	.00	− .16*			
Physical health	.00	− .35*			

*CFI* Comparative Fit Index; *RMSEA* root mean square error of approximation

*Significantly different from zero at the .05 level (two-tailed)

### Validity evidence. An extended structural model

The basic bifactor CFA solution used in the previous analyses was extended here to include three BSI scale scores (depression, anxiety, and total) that were used as external variables. Regarding the substantive results, psychological distress and depression are clearly the two factors that are most strongly and negatively correlated with the QoL factor. Anxiety was the scale that most closely correlated with the physical health factor. Since total BSI score is the sum of the other two subscale scores, a separate model was fitted for the former. The fit of the extended models was very similar to that of the base model; therefore, fit results are not reported here. The validity estimates are summarized in Table 5 and include disattenuated standardized regression coefficients (i.e., *ß* weights) and squared multiple correlations for each external variable. The first coefficient quantifies the predictive power of each factor (QoL and Physical Health) relative to the external variable. The squared multiple *R* quantifies the variance of the external variable that can be explained by both factors together.

**Table 5:** Validity assessment

Factor	Depression	Anxiety	BSI total
Quality of life	− .62**	− .56**	− .70**
Physical health	− .33**	− .51**	− .31**
*R*^2^	.49**	.58**	.59**

Structural standardized validity coefficients and multiple R^2^ between the EORTC factors and the BSI scores

**p < .001

### Normative data

To facilitate clinical interpretation by psychologists and oncologists, we have introduced a normative table to convert raw scores to *T*-scores and centiles. The overall quality of life (QOL) and physical health scores are interpreted as the higher the score, the better the quality of life and physical status, see Table 6.

**Table 6:** Scale table to convert raw scores to *T*-scores and centiles

Centile	QoL		Functional health		Centile
	Raw score	*T*-score	Raw Score	*T*-score	
99	93	66	97–100	61	99
97	92	65	–	–	97
95	91	–	–	–	95
93	90	64	–	–	93
91	89	63	–	–	91
90	88	62	–	–	90
88	87	61	–	–	88
86	86	60	–	–	86
83	85	59	94–96	59	83
79	84	–	–	–	79
73	82–83	57	93	57	73
71	81	56	–	–	71
68	80	–	92	57	68
64	79	55	90–91	56	64
61	78	55	–	–	61
58	77	54	86–89	55	58
56	76	52	–	–	56
51	75	52	85	53	51
48	72–74	50	81–84	52	48
40	69–71	48	78–80	50	40
34	66–68	46	75–77	48	34
27	64–65	44	71–74	46	27
22	61–63	42	68–70	44	22
17	59–60	40	64–67	42	17
15	58	39	63	39	15
11	57	38	57–59	37	11
10	51–56	37	49–56	35	10
5	35–50	33	31–48	31	5
1	0–34	20	0–30	20	1
Mean	72.7		80.9		
S.D	13.0		16.9		
Reliability	0.94		0.86		

## Discussion

The aim of this study was to appraise the psychometric properties of the Spanish version of the EORTC QLQ-C30 in a sample of patients with varying diagnoses. The results support the use of the EORTC scale to gauge quality of life in patients with cancer and confirm the stability of its construct in different types of tumor, which has also been corroborated by other studies [4, 32, 33]

As previously mentioned, the factorial structure found in this study is organized around two factors: Quality of life and Physical health. The first factor includes all the items on the scale, reflecting a plausible unidimensional structure, in which all the items exhibit suitable factor loadings and proper goodness-of-fit indicators and could reflect physical well-being in cancer patients. This unidimensional HRQL structure was similarly encountered by [18, 34] in which all the items are assumed to be latent variables of a single dimension and are a good indicator of HRQL [4, 32, 33]. In our series, items 29 and 30 that originally pertains to the overall health scale would be included in the general factor of our quality-of-life rating scale. Nevertheless, some authors exclude these items from the general factor because they believe that they compound the issues of multicolinearity of the scale [35, 36], whereas other authors include them in the physical health factor [37, 38].

The second factor would cluster *physical health*-related items (difficulty in taking a long or short walk, trouble doing strenuous/nonstrenuous activities, difficulty remaining seated, and symptoms de fatigue). It displays high reliability, consistency over time, and strong correlations with validity. This factor would coincide in part with the findings of [16]. This factor could be sensitive to the toxicity of the treatment, in our series, it would be sensitive to physical impairment in patients with breast cancer, who would receive more intensive treatment than the other tumors and that would negatively affect their physical status, and would exhibit more symptoms of fatigue.

In our study, both the structure, as well as the content of the EORTC items remain stable in terms of sex and tumor site, as supported by the multi-group confirmatory factor analysis results. In a sample of patients with hematological neoplasms, support was attained for partial scalar invariance for sex and disease, mainly on the physical functioning and emotional functioning [39] scales. Similar results were observed in a sample of individuals with different kinds of cancer [40], they found that the model achieved good fit except for the group of prostate cancer patients who were excluded from the analysis.

No differences were found for either quality of life or physical health on the basis of sex, but were found according to tumor site. Participants with breast cancer present better overall quality of life, but worse physical status. This may be due to patients with breast cancer being younger than those with colon cancer [41], as seen in our study. Differences in physical status may be attributable to breast cancer patients generally receiving three types of chemotherapy agents (anthracycline + cyclophosphamide + paclitaxel) that cause many side effects, while individuals with colon cancer only receive two (fluoropyrimidine+—oxaliplatin) that lead to less toxicity. Moreover, the cancer diagnosis appears to have a greater impact for women with breast cancer, with the presence of more anxiety and depression that would be explained, not only by age, but also by the significant, treatment-induced changes to their physical appearance, such as hair loss, mastectomy, or weight gain resulting from hormone therapy [41, 42]. In contrast, people with colon cancer displayed better quality of life than those with other types of tumors, such as gastric or pancreatic cancer. This could stem from the fact that colon cancer patients who undergo hemicolectomy associate fewer physical sequelae than those with gastrectomy (as occurs in cancer of the stomach) or pancreatoduodenectomies (in pancreatic cancer) [6].

Both factors proposed in this study for the EORTC structure were found to be strong, well-defined, replicable, and capable of providing highly accurate score estimates. Furthermore, the fidelity index reached values exceeding 0.91, which means that even the simple sum scores will generate accurate individual measures of both the quality of life and physical health dimensions. The reliability estimates of the sum scores evaluated by means of McDonal’s omega (ω) was excellent, with values of 0.94 for quality of life and 0.86 for the physical health dimension. Other studies have yielded similar results in which the values ranged from 0.74 to 0.95 [4, 32, 33].

As for time, the two factors are sensitive to change, specially quality of life. As expected, the sample scored lower for quality of life posttreatment. Analogous results were obtained in individuals with colon cancer [41], hematological cancer [39], and those with breast cancer [2]. Finally, both scales correlate closely with depression, anxiety, and psychological distress. In our study, the correlations reveal that the quality of life factor might reflect psychological distress and despair in patients, whereas the physical health factor would associate more closely with anxiety.

The study has a series of strengths and limitations. Its greatest strength is the analysis of the psychometric properties of the EORTC scale in a large sample of individuals with varying kinds of cancer, that enabled us to divide the sample randomly into two halves to be able to conduct exploratory and confirmatory analyses. Among the limitations, the use of a cross-sectional design, which precludes inferring the direction between the relations that were found. Causality should be examined in future studies. Second, this sample consisted of individuals with a heterogenous, localized of tumor. Third, the results cannot be generalized to patients with advanced tumors or cancer survivors. Finally, we must be cautious when interpreting these results, bearing in mind that all the subjects eligible to participate did so voluntarily, which may have introduced a self-selection bias.

To conclude, the Spanish version of the EORTC QLQ-C30 is a reliable and valid quality-of-life measure. We deem this version to be efficacious in the evaluation of patients with cancer undergoing treatment with chemotherapy with curative intent and that, moreover, is capable of identifying a subgroup of patients who may suffer striking impairment of their physical health.

## Data Availability

The database is available through a centralized web platform: www.neocoping.es.

## Footnotes

1 FACTOR software can be downloaded for free from the site: https://psico.fcep.urv.cat/utilitats/factor.

## Code availability

Patients are identified by an encrypted code known only to the local researcher.
